# Higher education teachers' digital competencies for a blended future

**DOI:** 10.1007/s11423-023-10211-6

**Published:** 2023-03-13

**Authors:** Sarah K. Howard, Jo Tondeur

**Affiliations:** 1 University of Wollongong, Northfields Ave., Wollongong, 2522 Australia; 2 Vrije Universiteit Brussel, Brussels, Belgium

**Keywords:** Digital competence, Teacher, Higher education

## Abstract

Welcome to this special issue, focussing on teachers’ digital competencies in Higher Education. The articles in this special issue explore the question of how digital competencies in higher education are conceptualized after the Great Online Transition, during and after the pandemic. A few of the topics addressed in the special issue are: new competency frameworks, the issue of disciplines and artificial intelligence and looks into the future of higher education learning and teaching. In this preface to the special issue, we first present a brief introduction to the context and the problem statement. We then provide a summary of each of the ten papers included in this special issue, we present how they are related and how each article makes a unique contribution to the main goal of the special issue. Finally, the implications are discussed together with suggestions for future research.

## Introduction to the special issue

The global COVID-19 pandemic catalyzed a significant ‘shift to digital’ in teaching and learning in higher education. Namely, this has been a result of the Great Online Transition (GOT) and global shift to online remote teaching (Howard et al., 2022). While this event has brought online teaching and digital technologies to the forefront of educational practice, the hope is that it has also opened the door to future innovation and change, new thinking and considerations about how we teach and learn. Therefore, it is now the time to consider how higher education teaching and learning has and potentially will continue to change as a result of this 1–2 year remote ‘experiment’. Yet, online teaching and learning is not new to higher education (Kebritchi et al., 2017; Scherer et al., 2021). Prior to this, and for almost two decades, institutions for higher education across the world have been gradually adopting online and blended learning practices (Singh & Thurman, 2019), but the nature and quality of online teaching and learning has been inconsistent (Bernard et al., 2014; Howard et al., 2020). As a result, what is expected for on-going digitally-supported learning in higher education has been unclear. This clouds how to best prepare higher education teachers with the necessary digital competencies needed to design and support future online and blended-learning experiences (Bolliger et al., 2019). This new focus presents a challenging but interesting opportunity for higher education institutions. They are in a position to consider how they might sustain and leverage online practices adopted during emergency remote teaching into blended learning—to a possible blended future for higher education.

An area of great interest will be how higher education teachers integrate and develop online practices adopted during the GOT as they return to their usual teaching, or in some cases where teaching is permanently changed. It is essential to understand which digital competencies will be important and what kind of support is needed to support on-gonig change in their teaching practice (Bruggeman et al., 2021). Typically, in terms of building teachers’ digital competencies, research has shown that higher education institutions commonly give consideration to technical issues, while pedagogical support is not often taken into consideration (Scherer et al., 2021). This is problematic, as technical competence is only part of the story. Considerations for pedagogical, social or well-being competencies are often missing from training. While there are a range of digital competency frameworks available to guide higher education practice, these should be reconsidered as expectations and approaches to teaching and learning are different after the GOT (McQuirter, 2020; Tondeur et al., 2017). Therefore, it remains largely undetermined: what do higher education teachers need to know about digital practice, teaching and learning? The answers to these questions will be critical in extending and sustaining rich online practices and developing blended learning after the GOT.

In response to issues arising from the GOT, in 2021 ETRD published the special issue “Shifting to Digital: Informing the Rapid Development, Deployment, and Future of Teaching and Learning” (Lin & Johnson, 2021). One of the key messages for future research arising from this work was a lack of clarity in what should be included to support and train teachers for the digital shift (Yuno, 2020). In the current special issue we addressed this gap in knowledge on higher education teachers' digital competencies and teaching practice to support changes after the GOT. An example of this is An’s (2021) response to Phillipsen et al.’s (2019) paper. Phillipsen et al. (2019) present a framework comprising what should be addressed for successful teacher professional development for online and blended learning. However, An (2021) notes that more research is needed to build a common understanding of the essential knowledge, skills, and competences for online teaching (cf. Bernard et al., 2014). It is in this space, and those spaces among other publications in the Shift to Digital, that was explored in the current special issue.

To explore teachers’ digital competencies as part of a post-GOT digital shift in higher education, the special issue unpacks competencies in relation to teachers’ pedagogical beliefs, policy planning, professional development, leadership, preparing students for future work, etc. The variety of studies also consider current issues related to the digital competencies of teachers in higher education, e.g. which digital competencies teachers in higher education exactly need to work in the post-GOT age, how to developed these competencies for specific teachers’ profiles, or how to develop teachers’ digital competencies that are of particular interest in view of the outcomes and challenges of the COVID-19 pandemic (e.g., flexibility, autonomy of students or inequities of learning online). These can also be extended to considering future learning designs, expectations of digital technology use, and equitable access to quality learning experiences.

## Positioning the articles

The first article, by Cook, Apps, Beckman and Bennett, first reviews empirical studies of emergency remote teaching to derive a conceptual frame for digital competence, which was then applied as a lens to analyze teaching in Australian universities. The findings provide a starting point for understanding digital competencies in Higher education in order to better understand the present and also to anticipate the future. More specifically, the authors suggest that institutions for higher education can better support the development of teachers’ digital competence through practical operationalisations that connect technical and pedagogical knowledge, make digital possibilities across modes of delivery more explicit, and should also protect the well-being of educators.

The second article brings the findings mentioned above together in a more generic framework of digital competencies for teachers in higher education, while extending the domains of pedagogical and technical knowledge to include empowering students. Tondeur, Howard, Van Zanten, Van der Neut, Gorissen, Kral and Uerz conducted a review to determine the state of digital competence research in general, similarities and differences between existing digital competence frameworks. Based on the outcomes of their review and the framework comparison, a framework was developed through expert meetings with policy makers and experts, and finally validated with practitioners. The resulting HeDiCom (Higher Education Digital Competence) framework provides guidance and clearer expectations of higher education teachers' digital competency which can guide innovation in practice beyond the GOT.

When considering how teachers have engaged in change, and how this relates to developing competencies, it is necessary to look at their experiences. How teachers themself experience their teaching and digital competencies has been explored in the third article. This qualitative study by González, Ponce and Fernández examined teachers’ experiences of teaching online during COVID-19 in Chile. The findings of their hybrid thematic analysis show that, despite problems faced by both pandemic and political disruption, there were several learned lessons: teachers employed an array of digital tools for maintaining content delivery and promoting interaction, deepened their understanding of course design and assessment, and developed an emphatic disposition to understand students’ situations in the context of Chilean Higher Education.

Students' experiences and their perceptions of teachers' digital competencies are also important, as they are a key factor in teachers' beliefs about teaching practice. Zhu investigated university teachers' digital competencies through the eyes of their students. This fourth article focused on a blended course on “Educational Technology” with theoretical and practical components in a Chinese university. The participants’ responses to the open-ended questions provided in-depth views of the factors contributing to university teachers’ digital competencies of blended teaching and how they can influence students’ learning. Also the participants’ expectations of future blended courses provided implications for teachers’ digital competencies in blended teaching in post-COVID-19 era from learners’ perspectives.

The fifth paper begins to take a more granular look at factors at play in the development of competencies. Trevisan, De Rossi, Christensen, Knezek and Smits explored factors shaping digital competencies. The authors present findings on higher education faculty’s reported dispositions and needs as they engaged with online teaching. They report on the findings of two surveys administered to higher education faculty worldwide during two time periods within the COVID-19 pandemic. The surveys inquired about internal (e.g., enthusiasm and resolutions) and external (i.e., support) facets of faculty’s perception of online teaching at two time periods. The findings reveal different patterns of dispositions and different uses of technological affordances to foster online learning. These patterns were also found to change over time, highlighting different conditions possibly enabling or hindering the development of competencies for online teaching and learning.

The research by Sang, Wang, Li, Xi and Yang deepens our understanding of developing competencies. They investigated the relationship between three specific factors in a Chinese normal university: digital competence, effort expectancy and teachers’ work engagement. The results of their structural equation modeling indicated that Higher Education teachers’ digital competence positively and significantly correlated with their work engagement and their effort expectancy. Overall, the findings imply that higher education teachers’ digital competence is an important motivator for their work engagement.

However, teachers' work is also strongly dictated by their specific disciplinary area. In the next paper, Starkey, Yates, Lundqvist, Ormond and Syvester explored the relationship between disciplines and higher education teachers’ digital competencies for future-focussed digitally infused undergraduate programmes. Their case studies identified how disciplinary culture, context, and technology influence pedagogical practice and digital competencies needed to teach in undergraduate programmes in New Zealand (e.g., Applied Statistics, Critical Indigenous Studies, Geography). They conclude that higher education teachers require digital competencies that align with disciplinary culture and the specific technologies available.

The special issue then begins to look forward to competencies and near-future changes in higher education teaching and learning. Tsz Kit Ng, Ka Lok Leung, Jiahong Su, Chi Wui Ng, and Kai Wah Chu consider the nature of Artificial Intelligence digital competencies. In their concept paper, they first explored the opportunities and challenges of employing AI systems and how they can enhance teaching, learning and assessment. To align with generic digital competence frameworks, they complemented the DigCompEdu framework and revised the P21’s framework to accommodate AI technologies in order to provide an overview of the necessary AI digital competencies.

To present which teachers’ digital competencies are needed is an important first step in the context of Higher Education. The Dexter article takes a next step and can serve as guidance to organize faculty decision making. Her post-hoc analysis provided principles to capture how participants in each study linked any ICT competencies to overall coherent ICT integration competence. This reasoning shifts more responsibility for setting directions and crafting aligned professional learning to the organization in a way that exceeds the “help desk” model, to also leverage and work with any university units concerned with quality in teaching and learning.

Finally, the conceptual paper by Markauskaite, Carvalho and Faws explored the role of higher education teachers' digital competencies to postdigital capabilities. In their future-oriented paper, they consider what it will take to be a teacher in a sustainable university and emerging trends at three levels of the educational ecosystem—global developments, teachers’ local practices, and daily activities. The authors argue that there is a need to move beyond person-centric theorisations of teacher digital competencies towards more holistic, ecological conceptualisations. It requires higher education teachers’ agency and critical engagement with a future-oriented, sustainable university mission, which is at its core postdigital.

## Lesson learned and directions for future research

It is critical that change momentum from remote teaching during the pandemic and the GOT is not lost in higher education. Never before has there been such a large-scale change initiative across education, which has resulted in this kind of dramatic reconsideration of teaching and learning in higher education. Universities have improved infrastructure, teachers and students have both upskilled, and everyone has been exposed to new practices. The articles presented in this special issue are all looking at how we can better understand teachers' digital competencies, which can support university policy, training and support structures to build on this strange and powerful event. Specifically, there is a significant need in the field for an understanding of where Higher Education teachers are currently with their digital competencies: what are teachers' doing well and where do they feel confident, and where is more work needed?

Moving from this point, research is needed to further understand how competency frameworks are implemented, if they are fit for purpose, and their validity and relevance in different contexts. As we look into the future, digital competencies will only become more complex, particularly with growing pressures to integrate artificial intelligence and ways of working rapidly changing. Higher Education has not generally operated within a coherent body of accepted practices for teaching and learning, and this is not necessarily needed. However, digital technologies are changing and will continue to change how individuals and organizations interact, work, teach, learn and create knowledge. Better understanding of competencies needed for fruitful engagement, for individuals to exert agency and thrive, is sorely needed at this time. This special issue presents a first step to opening up this problem space in the field.
